# Delignification of miscanthus using ethylenediamine (EDA) with or without ammonia and subsequent enzymatic hydrolysis to sugars

**DOI:** 10.1007/s13205-015-0344-z

**Published:** 2016-01-11

**Authors:** Sasisanker Padmanabhan, Philippe Schwyter, Zhongguo Liu, Geoffrey Poon, Alexis T. Bell, John M. Prausnitz

**Affiliations:** 1 Energy Biosciences Institute, University of California, Berkeley, CA 94720-1462 USA; 2 Department of Chemical and Biomolecular Engineering, University of California, Berkeley, CA 94720-1462 USA; 3 Praj Matrix R & D Center, Division of Praj Industries Ltd, Pune, 412115 India

**Keywords:** Ethylenediamine (EDA), Delignification, Miscanthus, Enzymatic hydrolysis, Ammonia, Auto-hydrolysis

## Abstract

Pretreatment of miscanthus is essential for efficient enzymatic production of cellulosic ethanol. This study reports a possible pretreatment method for miscanthus using aqueous ethylenediamine (EDA) for 30 min at 180 °C with or without ammonia. The mass ratio of miscanthus to EDA was varied from 1:3, 1:1, and 1:0.5, keeping the mass ratio of miscanthus to liquid (EDA + Water) constant at 1:8. The ammonia-to-miscanthus ratio was 1:0.25. After pretreatment with a ratio of 1:3 miscanthus to EDA, about 75 % of the lignin was removed from the raw miscanthus with 90 % retention of cellulose and 50 % of hemicellulose in the recovered solid. Enzymatic hydrolysis of the recovered solid miscanthus gave 63 % glucose and 62 % xylose conversion after 72 h. EDA provides an effective pretreatment for miscanthus, achieving good delignification and enhanced sugar yield by enzyme hydrolysis. Results using aqueous EDA with or without ammonia are much better than those using hot water and compare favorably with those using aqueous ammonia. The delignification efficiency of EDA pretreatment is high compared to that for hot-water pretreatment and is nearly as efficient as that obtained for aqueous-ammonia pretreatment.

## Introduction

Economic and environmental concerns about the continued use of fossil fuels have prompted a search for alternative fuels using sustainable lignocellulosic biomass feedstock (Carroll and Somerville 2009). Conversion of abundant and renewable lignocellulosic biomass to bioethanol as a transportation fuel provides a possible option to reduce greenhouse gas emissions and fossil fuel consumption (Jordan et al. 2012). Miscanthus is a suitable biomass feedstock because it has high carbohydrate density and low fertilizer requirements for growth (Padmanabhan et al. 2012; de Vrije et al. 2009; Carroll and Somerville 2009; Brosse et al. 2012).

The primary constituents of miscanthus biomass are cellulose, hemicellulose, and lignin (Brosse et al. 2010, 2012; de Vrije et al. 2009; Carroll and Somerville 2009). For efficient production of cellulosic biofuels, miscanthus must be pretreated to overcome the barriers that hinder the hydrolysis of cellulose and hemicellulose to fermentable sugars (Kumar et al. 2009; Rodriguez et al. 2011; Shill et al. 2011; Alvira et al. 2010; Agbor et al. 2011; Cao et al. 2012). To facilitate enzymatic hydrolysis, an effective pretreatment must remove a significant fraction of lignin and perhaps, reduce the crystallinity of cellulose (Kumar et al. 2009; Shill et al. 2011). By decreasing the adherence of lignin to cellulose, and by disrupting the cellulose crystal structure, the barriers to hydrolysis are reduced; hydrolytic enzymes can then access the carbohydrates more easily (Geng and Henderson 2012; Klein-Marcuschamer et al. 2010).

A wide variety of pretreatments has been studied leading to various degrees of success (Blanch et al. 2011; Sousa et al. 2009; Kim et al. 2011). Of these, organosolv pretreatment has long been employed for delignification (Brosse et al. 2009, 2010). Organic solvents, with a little amount of inorganic acid as catalyst, can achieve a good degree of delignification (El Hage et al. 2009). Various studies in the paper-and-pulp industries have suggested that a combination of alkali and an organic solvent can yield a cellulose-rich pulp with a low amount of lignin (Abbot and Bolker 1982; Cochaux et al. 1995). Taking some clues from these studies, several research groups have employed combination of ammonia or methyl amine with organic solvents or ionic liquids for delignification of biomass (Cho et al. 2008; Cochaux et al. 1995; Rodriguez and Jimenez 2008; Sarwar et al. 2001; Abbot and Bolker 1982). Also, our recent work on delignification of miscanthus using a combination of EDA with organic solvents or ionic liquids suggests that EDA helps to cleave the lignin-carbohydrate link leading to good removal of lignin (Padmanabhan et al. 2012). EDA provides an added advantage with respect to higher alkalinity (pH > 12), which is favorable for the removal of lignin from miscanthus.

Pretreatment is the most expensive step in the overall production of bioethanol fuel (Klein-Marcuschamer et al. 2010; Tao et al. 2011). Therefore, it is desirable to minimize the loading of reagents or solvents in biomass treatment while nevertheless achieving a high sugar yield. To determine the viability of EDA in the pretreatment of miscanthus, we examine the effects of low and high EDA loadings to miscanthus (0.5:1, 1:1, 3:1). In addition to reporting EDA-based-pretreatment results, this work also gives a brief comparison with two well-known pretreatment methods: auto-hydrolysis (hot water) or aqueous ammonia.

## Materials and methods

### Raw materials and feedstock

Miscanthus (*Miscanthus* × *giganteus*) samples, obtained from the University of Illinois at Urbana-Champaign, were milled to 4-mm particles using a rotary mill (Hetsch). These particles were air-dried and stored in a sealed container. The moisture content of the miscanthus was 6.1 % determined using a halogen-moisture analyzer (Mettler-Toledo). Table 1 shows the composition of the raw untreated *Miscanthus* × *giganteus* determined using the procedures recommended by the National Renewable Energy Laboratory (NREL) and other recent publications (Sluiter et al. 2010a, b; Templeton et al. 2010; Ibáñez and Bauer 2014).

**Table 1:** Contents of individual components (weight %) of raw miscanthus and recovered solid miscanthus after pretreatment using solvents. For comparison, results are shown for untreated miscanthus

Solvent	Recovery (%)	Miscanthus-to-solvent ratio^a^	Temperature (°C)	Cellulose content in the recovered miscanthus (%)	Hemicellulose content in the recovered miscanthus (%)	Lignin content in the recovered solid (%)	Others (Ash, salts, extractives^a^) in the recovered (%)
Untreated	–	–	–	41.5	24.5	26	6
Hot water	73	1:8	180	5.4	13.4	21.5	6.4
10 % aqueous ammonia	64	1:8	180	60.8	20.2	14.1	8.2
30 % aqueous ammonia	60			61.3	16.5	10.5	7.5
Aqueous EDA + ammonia^a^	62	1:3	180	61.4	16.4	9.4	7.4
	65	1:1		59.4	18.2	11.4	8.4
	70	1:0.5		57.4	20.3	13.5	6.6
Aqueous EDA without ammonia^a^	63	1:3		58.5	18.2	10.3	6.7
	65	1:1		56.4	21.3	11.2	9.5
	71	1:0.5		56.2	22.4	14.5	8.1

^a^For aqueous EDA (EDA + Water), the total solvent-to-miscanthus ratio is 8:1 while miscanthus-to-EDA ratio is also indicated in the table. Miscanthus to ammonia is approximately 1:0.25, calculated on basis of autoclave volume, temperature and pressure. Extractives are soluble sugars, non-structural sugars and others which were extracted in hot water/ethanol solution at 80 °C for 6 h

EDA was purchased from Sigma-Aldrich (USA) with a purity of 99.5 % and used as received. Compressed anhydrous ammonia with a purity of 99.99 % was purchased from Praxair, USA.

Cellobiase enzyme from *Aspergillus niger* and Cellulase enzyme produced from *Trichoderma reesei ATCC 26921* were obtained from Sigma-Aldrich (St. Louis, MO). All enzymes were stored at 4 °C until use for hydrolysis. Cellulase and cellobiase (β-glucosidase) activities were 700 and 250 U/g, as provided by the supplier.

Citrate buffer was made from anhydrous citric acid (Fisher Scientific) and sodium citrate dehydrate (Research Organics Inc). pH was measured using a Mettler-Toledo pH meter. Sodium azide, 0.5 % (w/v), was obtained from Ricca Chemical Company and used as received.

### Pretreatment procedure

#### EDA pretreatment

**Fig. 1:**
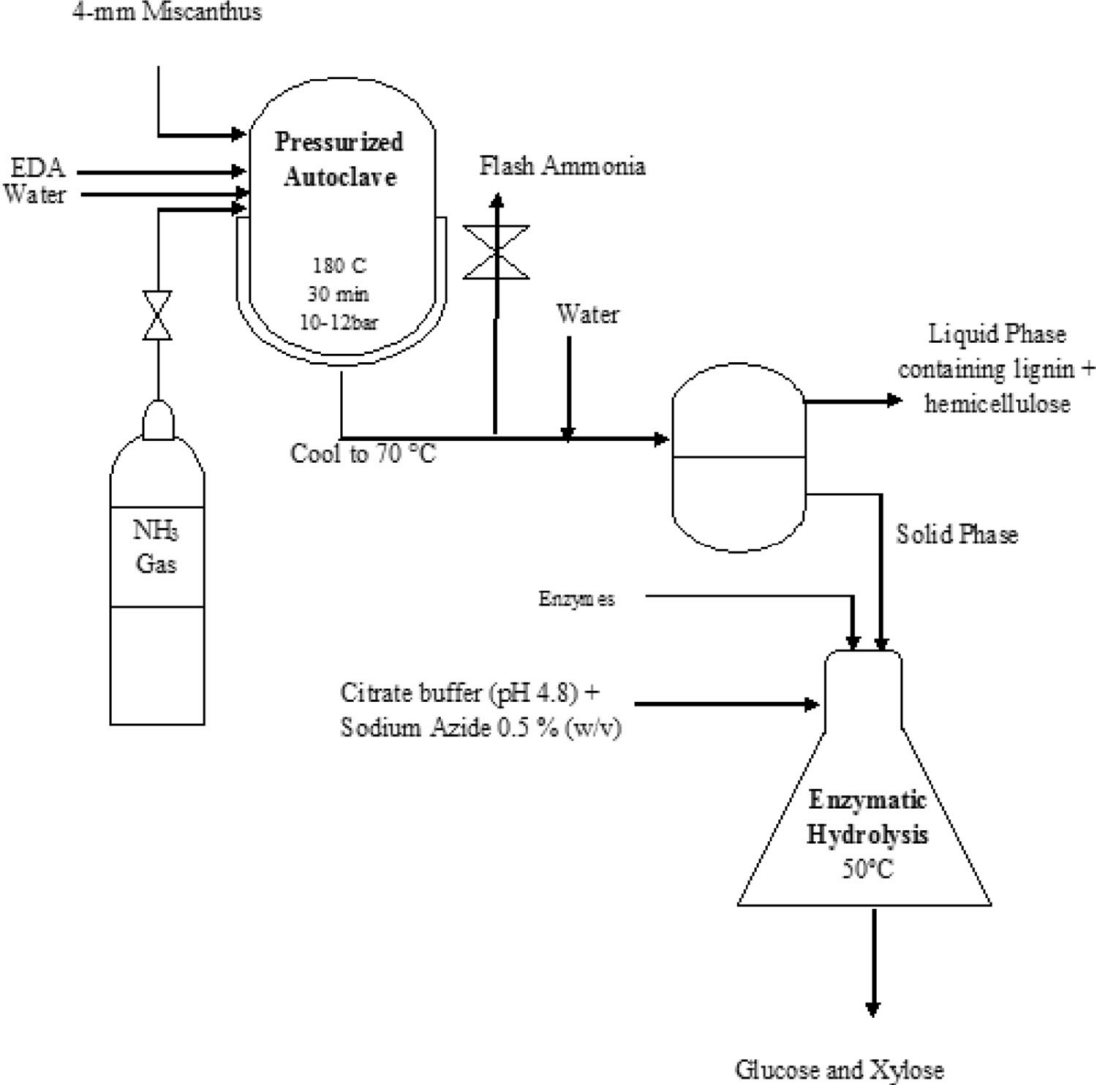
Schematic diagram of pretreatment process

Figure 1 shows a schematic pretreatment diagram. Miscanthus and EDA were placed inside a batch pressure reactor (Moline Parr Instruments) with a capacity of ca. 20 cm^3^. One gram of miscanthus was added to a variable amount of EDA to achieve the desired miscanthus-to-EDA ratio for a particular pretreatment. Deionized milli-Q water was subsequently added until the total net weight of the solid–solvents mixture was 8 g. If the pretreatment used gaseous ammonia, the mixture was pressurized with 10 bar ammonia.

The reactor was placed in a temperature-controlled oil bath at 180 °C for 30 min. The reactor was then removed from the bath and cooled to room temperature. If the reactor contained ammonia, the ammonia was flashed after cooling.

The slurry was filtered. The liquid phase (EDA, water and dissolved biomass) was collected using vacuum filtration and stored for composition analysis. The remaining solid phase (recovered solid) was washed with water until pH 7 was achieved.

For each pretreatment condition, the pretreatment was replicated five times. Chemical composition analysis was done in triplicate, and enzymatic hydrolysis was done in duplicate, all with good reproducibility.

#### Auto-hydrolysis

Auto-hydrolysis experiments were carried out in the batch reactors mentioned in the previous section. Miscanthus-to-water ratio was set to 1:8 (w/w) and the pretreatment was carried out for 30 min at 180 °C. After 30 min, the slurry was cooled to room temperature, filtered using Whatman 41 filter paper to separate the liquid from the solid. The solid was dried in an oven at 105 °C for 12 h. The liquid was stored for chemical composition analysis. The chemical composition analysis of the solid followed the NREL protocol summarized here and in our previous publications (Padmanabhan et al. 2011; Rodriguez et al. 2011; Sluiter et al. 2010a, b).

#### Aqueous Ammonia

1 g of 4-mm-particle miscanthus and 10 gm of 5 or 30 % aqueous ammonia were placed in the pressurized autoclave. After 30 min at 180 °C, the miscanthus + ammonia slurry was cooled to room temperature prior to solid–liquid separation by filtration.

### Chemical composition analysis of untreated and pretreated solid miscanthus

Analysis of untreated and pretreated miscanthus followed the protocols mentioned in recent publication of Ibáñez and Bauer (2014). This method is essentially a modification of NREL protocol to accommodate smaller size samples (Sluiter et al. 2010a, b; Templeton et al. 2010). The details of the protocols can be found in the publication of Ibáñez and Bauer (2014). A brief summary of the method employed is summarized here. The original and the recovered solid were dried in an oven at 105 °C at atmospheric pressure overnight until its dry weight did not change. The mass of the solid was then measured using a Mettler-Toledo electronic weighing balance with a precision of ±0.0001 g.

To obtain a material balance, amounts of cellulose, hemicellulose, and lignin present in the recovered solid were determined based on products obtained from acid hydrolysis. Three 50-mg samples of the dried solid were placed in separate 17-mL glass autoclave tubes. Half a milliliter of 72 % H_2_SO_4_ aqueous solution was then added to each tube. The hydrolysis was allowed to proceed for 1 h at room temperature. Brief vortexing with a Fisher Scientific digital vortex mixer was done every 10 min during the reaction to improve solid–liquid contact. After 1 h, 14 mL of water were added to reduce the acid concentration to 4 wt%. The tubes were then autoclaved at 121 °C and 1.38 bar for 1 h. The contents of the tube were cooled prior to filtration using glass filters (Millipore, Ireland).

The liquid phase from acid hydrolysis contained sugars and minor amounts of degradation products, mainly furfural and 5-hydroxymethylfurfural (HMF), in addition to any acid-soluble lignin. The solid residue contained acid-insoluble lignin and ash.

A one-milliliter aliquot of the liquid filtrate was filtered again using a 200-nm filtering disc and then analyzed using a Shimazdu HPLC equipped with a 300-mm × 7.8 mm Aminex HPX-87H column (Bio-Rad Laboratories) and a refractive-index detector. The mobile phase was 0.001 N–H_2_SO_4_ aqueous solution at a flow rate of 0.6 mL/min. The injection volume was 20 μL. The concentrations of sugars and degradation products were determined using previously established calibration curves.

The cellulose content in the original or recovered miscanthus was calculated from the concentrations of glucose and HMF. The hemicellulose content was calculated from the concentrations of xylose, arabinose, acetate, and furfural. These calculations followed the standard NREL analytical procedure.

The acid-soluble-lignin content is determined by analyzing the ultraviolet absorbance of the liquid filtrate at 205 nm using an Agilent 8453 UV–Vis spectrophotometer. An aliquot of the filtrate was diluted as needed (such that the absorbance was in the range 0.3–0.7) and placed in quartz cuvettes. The percent acid-soluble lignin was calculated from

1 $${\text{Percent}}\;{\text{acid}}\;{\text{soluble}}\;{\text{lignin}} = \frac{{({\text{Absorbance}}\;{\text{at}}\; 2 0 5\;{\text{nm}}) \times ({\text{Dilution}}\;{\text{factor}}) \times ({\text{Volume}}\;{\text{of}}\;{\text{filtrate,}}\; 1 4. 5\;{\text{mL}})}}{{({\text{Culette}}\;{\text{path}}\;{\text{length,}}\; 1\;{\text{cm}}) \times ({\text{Absorptivity}}\;{\text{at}}\; 2 0 5\;{\text{nm}}) \times ({\text{Mass}}\;{\text{of}}\;{\text{solid}}\;{\text{hydrolyzed}})}} \times 100$$

The acid-insoluble lignin is the difference between the dry weight of solid residue from acid hydrolysis and its weight after it was ashed. To calculate the dry weight, the solid residue after filtering the autoclaved contents was oven-dried at 105 °C overnight; this weight was recorded. The dried solid was then placed in aluminum pans of known mass. The pan is then placed in a furnace and ashed by raising the temperature to 575 °C for 3–4 h. The aluminum pans had previously been ashed using the same thermal treatment to avoid errors due to residual organic substances on the pan. The percent acid-insoluble lignin was calculated from

2 $${\text{Percent acid insoluble lignin}} = \frac{{\left( {\text{Mass before ashing}} \right) - \left( {\text{Mass after ashing}} \right)}}{{\left( {\text{Mass of solid hydrolyzed}} \right)}} \times 100$$

The total lignin content is the sum of the acid-soluble and acid-insoluble lignin.

### Enzymatic hydrolysis

The washed recovered solid was placed in a 125 mL Erlenmeyer flask. For 1 g of recovered biomass, 50 mL of citrate buffer solution (50 mM, 4.8–5.0 pH), 4.00 mL sodium azide solution (0.5 % w/v), was added to the Erlenmeyer flask. The purpose of sodium azide is to inhibit any microbial growth during enzyme hydrolysis. Cellulase and β-glucosidase were added at a loading of 20 FPU (Filter Paper Unit) per gram of cellulose. Cellulase measurement units (FPU) were determined according to the procedure described by Ghose (1987). Enzymatic hydrolysis was conducted at 50 °C in an Innova 44-series incubator with shaking at 150 revolutions per minute. The pH of the system was maintained in the range of 4.8–5.0, which is considered as the optimal conditions for cellulase enzymes (Ghose 1987). Aliquots of 200 μL of supernatant were taken after 72 h. Each aliquot was diluted by a factor of two and analyzed using a Shimadzu HPLC.

Conversions of cellulose to glucose and hemicellulose to xylose are calculated using Eqs. 3 and 4. We report conversions for the pretreated solid miscanthus, excluding what dissolved in the aqueous phase.

3 $${\text{Percent cellulose conversion }} = \left[ {\frac{{{\text{Glucose concentration}}\left( {\frac{\text{mg}}{\text{mL}}} \right) \times {\text{hydrolysis volume}}\left( {\text{mL}} \right) \times 0.9}}{{{\text{Cellulose}}\left( {\text{mg}} \right){\text{in the pretreatedmiscanthus}}}}} \right] \times 100$$

In the above equation, the constant 0.9 accounts for hydration of cellulose to produce glucose.

4 $${\text{Percent hemicellulose conversion}} = \left[ {\frac{{{\text{Xylose concentration}}\left( {\frac{\text{mg}}{\text{mL}}} \right) \times {\text{hydrolysis volume}}\left( {\text{mL}} \right) \times 0.88}}{{{\text{Hemicellulose}}\left( {\text{mg}} \right){\text{in the pretreatedmiscanthus}}}}} \right] \times 100$$

In Eq. (4), the constant 0.88 accounts for hydration of hemicellulose to produce xylose.

Additional production of glucose and xylose (not studied here) can be obtained from cellulose and hemicellulose in aqueous EDA stream.

## Results and discussion

### Effect of EDA with and without ammonia on delignification of miscanthus

Delignification of miscanthus after pretreatment is calculated from:

5 $${\text{Percent delignification = }}\left( {\frac{{{\text{Lignin content in untreated solid - }}\left( {{\text{lignin content in pretreated solid}} \times \frac{\text{recovery}}{ 1 0 0}} \right)}}{\text{lignin content in untreated solid}}} \right) \times 1 0 0$$

**Fig. 2:**
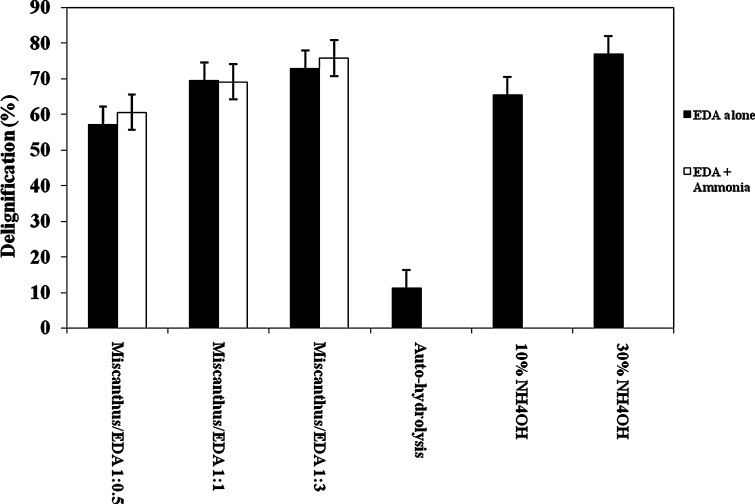
Comparison of delignification of miscanthus from different pretreatment methods at 180 °C for 30 min. Uncertainty in delignification is ±5 %

Figure 2 and Tables 1 and 2 show the effect of EDA on delignification of miscanthus with and without ammonia. Compared to hot-water pretreated miscanthus, addition of EDA leads to significant lignin removal. As expected, increasing concentration of EDA leads to higher delignification. When the miscanthus-to-EDA mass ratio is 1:0.5, nearly 58 % delignification is achieved. Higher miscanthus-to-EDA ratio (1:3) raises lignin removal to approximately 72 %. Because EDA has appreciable hydrogen basicity, it provides delignification larger than those using conventional polar solvents (Ishikura 2011; Padmanabhan et al. 2012). Our previous work on solubility studies of miscanthus using COSMO-RS (conductor-like screening model–real solvents) indicated that ammonia, amines and EDA favor interactions with the phenolic-OH group of lignin due to their strong hydrogen-bond basicity (Rodriguez et al. 2011; Padmanabhan et al. 2011). This basicity contributes to better solubilization and removal of lignin.

**Table 2:** Percent of cellulose, hemicellulose and lignin transferred to liquid phase after pretreatment

Solvent	Temperature (°C)	Miscanthus-to -solvent ratio	Cellulose transferred to liquid phase (%)	Hemicellulose transferred to liquid phase (%)	Lignin transferred to liquid phase (%)
Water	180	1:8	4.6	42.5	11.2
10 % aqueous ammonia	180	1:8	5.2	46.5	65.5
30 % aqueous ammonia			11.4	58.4	76.9
Aqueous EDA + Ammonia^a^	180	1:3	9.36	54.63	75.72
		1:1	9.62	52.71	69.13
		1:0.5	9.33	36.79	60.63
Aqueous EDA without ammonia^a^		1:3	12.3	52.23	72.96
		1:1	11.82	42.31	69.67
		1:0.5	7.4	33.73	57.10

^a^For aqueous EDA (EDA + Water), the total solvent-to-miscanthus ratio is 8:1 while miscanthus-to-EDA ratio is also indicated. Ratio of miscanthus to ammonia is approximately 1:0.25, calculated on basis of autoclave volume, temperature and pressure

Figure 2 also shows that delignification of miscanthus with EDA and ammonia is nearly the same as that of EDA without ammonia. About 75 % of lignin is removed upon addition of ammonia to 1:3 mass ratio of miscanthus to EDA. Because there is no significant improvement in delignification upon addition of ammonia, EDA alone appears to be sufficient for good lignin removal.

### Comparison of EDA delignification with delignification using hot water or aqueous ammonia

Figure 2 shows the extent of delignification of miscanthus using: (a) auto-hydrolysis, (b) aqueous ammonia and (c) EDA (with and without ammonia). The extent of delignification for EDA pretreatment is nearly same as that using aqueous ammonia for pretreatment of miscanthus and other biomass feedstock (Kurakake et al. 2001; Wyman et al. 2009; Boonmanumsin et al. 2012; Kim et al. 2009). Delignification results obtained here are comparable to those reported elsewhere for alkaline pretreatment (Wang et al. 2004, 2012; Elander et al. 2009; Gupta and Lee 2010; Tao et al. 2011; Park et al. 2010). Our previously reported work on pretreatment of miscanthus using aqueous ammonia (10, 20 and 30 %) achieves nearly 75 % delignification (Liu et al. 2013).

Figure 2 shows that both aqueous ammonia and EDA provide similar delignification despite different chemical mechanisms. For aqueous ammonia, the OH^−^ nucleophile is responsible for the breaking of lignin–carbohydrate interactions, whereas for EDA the interaction mostly follows from the two NH_2_ groups present in the diamine.

Figure 2 suggests that hot-water pretreatment alone is not sufficient to remove a significant amount of lignin. Only 10–12 % delignification was achieved with the hot-water pretreatment at 180º C, consistent with results reported by others (Ingram et al. 2011b; Wyman et al. 2011). Ingram et al., and Wormeyer et al., have compared lignin removal efficiency of hot water versus organosolv pretreatment for wheat straw feedstock (Wormeyer et al. 2011; Ingram et al. 2011a, b). Their studies confirm that organosolv pretreatment is more efficient than hot water for lignin removal.

Low and high loading of EDA gives delignification similar to that reported in our previous work on binary solvent mixtures of EDA with a polar organic solvent or an ionic liquid (Padmanabhan et al. 2012). Previous work, however, used higher loadings of EDA and a smaller miscanthus particle size (80 μm). In this study, with an eye toward economic process design, we have explored delignification using low-to-high loadings of EDA with larger miscanthus particle size (4 mm).

Delignification of miscanthus in aqueous EDA may be due to a reaction that decreases the molecular weight of lignin (Helmy and Aboustate 1993). Several alkaline pretreatment studies suggest that addition of alkali prevents repolymerization of lignin oligomers (Jahan and Farouqui 2000; Wang et al. 2010; Sarwar et al. 2001; Rodriguez and Jimenez 2008). Addition of ammonia or EDA to hot water leads to better solubilization of lignin in the liquid alkaline phase (Jahan and Farouqui 2002; Sun et al. 2010).

### Enzymatic hydrolysis of EDA-pretreated miscanthus

**Fig. 3:**
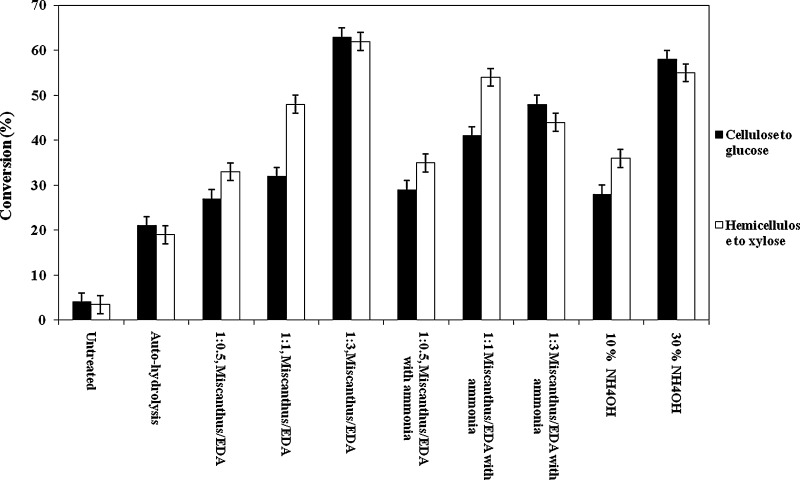
Percent conversion of cellulose and hemicellulose to corresponding glucose and xylose following enzymatic hydrolysis of pretreated miscanthus. Conversions are based on the recovered solid miscanthus. Refer Table 1 for the composition of recovered solid miscanthus. Uncertainty in conversion is ±3 %

Figure 3 shows results of enzymatic hydrolysis following three different pretreatments. For comparison, Fig. 3 also presents results for enzymatic hydrolysis of raw untreated miscanthus. As expected, untreated miscanthus shows very low conversion of carbohydrates to fermentable sugars, even after 1 week. Our studies show, once again, that pretreatment is necessary to break the recalcitrance of miscanthus for enzymatic hydrolysis to sugars.

Auto-hydrolysis pretreatment without alkali gives only minor conversion of cellulose to glucose after 72 h. The conversion is only about 20 %. This low conversion indicates that alkali is necessary to achieve the structural changes in cellulose required for significant delignification.

Pretreatment of miscanthus with EDA at various loadings with or without ammonia gives much better enzymatic hydrolysis compared to that for untreated or hot-water pretreated miscanthus. There is a strong correlation relating higher EDA loadings for improved conversion to glucose and xylose. A higher ratio of miscanthus to EDA corresponds to higher pH. As the EDA loading increases from 0.5:1 to 3:1(keeping temperature constant), enzymatic conversion improves from 27 to 63 % after 72 h.

The observed increase in conversion to sugars probably follows from higher delignification that enhances accessibility of enzymes to cellulose and hemicellulose. However, removal of lignin alone may not be sufficient for higher enzymatic conversions; probably, it is also important to bring changes in the structure of cellulose and to remove hemicellulose. Addition of ammonia to EDA does not appear to produce significant increase in sugar yield after enzyme hydrolysis.

The Tables 1 and 2 show that about one-half of the hemicellulose is retained in the recovered solid while the other half is dissolved in the liquid phase. Hemicellulose in the recovered solid may limit the accessibility of Cellulase enzymes. Hemicellulose oligomers provide a barrier to Cellulase enzymes, as shown by Wymann and coworkers (Qing and Wyman 2011; Kumar and Wyman 2009). Their results show that either addition of supplemental Xylanase enzymes or removal of hemicellulose raises the conversion of cellulose to glucose.

Although special hemicellulase enzymes were not added in our work, we nevertheless observed production of xylose. Some hemicellulase (xylanase) enzymes are present in the Celluclast enzyme cocktail even after purification, as indicated by the supplier. Similar to that of glucose, xylose yield rises upon increasing the loading of EDA. Upon raising EDA loading from 0.5:1 to 3:1, hemicellulose to xylose conversion increases from 33 to 55 % after 72 h. For auto-hydrolysis, hemicellulose to xylose conversion rate is same as that of cellulose to glucose. The presence of hemicellulose and lignin may perhaps be limiting the conversion rate. On the other hand, for ammonia alone treatment as well as for EDA with and without treatment, hemicellulose to xylose conversion rate raises with increase in the loadings of either ammonia or EDA. This perhaps confirms the observation that removal of hemicellulose as well as lignin may help achieve enzyme conversion.

## Conclusion

EDA provides an effective pretreatment for miscanthus, leading to good lignin removal and enhanced sugar yield by enzyme hydrolysis. Up to 75 % of lignin is removed leading to a yield of 63 % glucose and 62 % xylose from the recovered solid. The delignification efficiency of EDA pretreatment is high compared to that for hot-water pretreatment and is nearly as efficient as that obtained for aqueous-ammonia pretreatment. Addition of ammonia to EDA does not increase enzymatic conversion to sugars despite more removal of lignin and hemicellulose. Increasing EDA loading provides improved conversion of cellulose to sugars. Chemical composition analysis shows that better lignin removal improves sugar yields. About one-half of hemicellulose is removed from the solid during pretreatment; it is dissolved in the liquid phase, where, following adjustments to lower pH, it can be converted to xylose.

## Abbreviations

EDA: Ethylene diamine
NREL: National Renewable Energy Laboratory
HMF: 5 hydroxymethyl furfural
FPU: Filter paper units
HPLC: High-pressure liquid chromatography
COSMO-RS: Conductor-like screening model–real solvents
